# Supplementary material for: Cataract Surgery in Elderly Subjects with Heterozygous Familial Hypercholesterolemia in Prolonged Treatment with Statins
Source: J Clin Med. 2021 Aug 8;10(16):3494. doi: 10.3390/jcm10163494 (PMC8397196; doi:10.3390/jcm10163494)
Supplement: Supplementary file 1 [file jcm-10-03494-s001.zip › jcm-1305144-supplementary.pdf]

Supplemental Table. Clinical and laboratory characteristics of controls and cases according to previous cataract surgery.

**Mean (SD) / proportion [n]**		**Controls N=93**					**Cases N=112**			
	**N**	**No cataract surgery= 78**	**N**	**Cataract surgery= 15**	**P-value**	**N**	**No cataract surgery= 83**	**N**	**Cataract surgery= 28**	**P-value**
Age (years)	78	69.2 (7.1)	15	73.9 (7.3)	0.028	83	71.0 (6.2)	28	74.0 (6.8)	0.026
Weight (Kg)	78	74.9 (13.2)	14	81.3 (19.2)	0.235	82	71.6 (12.7)	27	73.6 (16.0)	0.668
Lp(a) (mg/dl)	8	46.1 (41.9)	2	48.0 (21.2)	0.518	47	60.0 (61.5)	12	51.2 (56.2)	0.943
SBP (mmHg)	77	136.9 (14.3)	15	135.5 (18.4)	0.512	81	134.1 (16.7)	28	134.9 (17.9)	0.550
DBP (mmHg)	77	80.6 (8.7)	15	73.7(12.8)	0.020	81	76.6 (9.2)	28	79.1 (11.0)	0.105
BMI (Kg/m2)	78	28.8 (4.5)	14	29.6 (6.1)	0.804	82	28.3 (3.9)	27	28.2 (4.5)	0.759
Untreated total cholesterol (mg/dl)	78	230.0 (39.7)	15	191.3 (48.3)	0.009	82	395.2 (71.9)	28	398.2 (78.9)	0.710
Untreated triglycerides (mg/dl)	78	150.0 (126.3)	15	123.3 (68.8)	0.245	83	138.8 (75.6)	28	143.5 (80.6)	0.851
Untreated HDLc (mg/dl)	77	58.0 (15.6)	14	50.7 (12.6)	0.464	83	56.2 (13.7)	28	54.7 (13.5)	0.841
Untreated LDLc (mg/dl)	75	141.0 (30.1)	15	123.3 (36.5)	0.170	82	313.9 (70.7)	28	314.9 (75.9)	0.899
Packages/day · years	76	11.5 (20.9)	14	35.0 (36.9)	0.007	81	9.6 (22.5)	26	10.7 (19.3)	0.968
Hypertension, % [n]	78	53.8 [42]	15	66.7 [10]	0.940	83	54.2 [45]	28	57.1 [16]	0.992
Type 2 Diabetes, % [n]	78	10.3 [8]	15	33.3 [5]	0.137	83	18.1 [15]	28	32.1 [9]	0.162
Previous cardiovascular disease, % [n]	78	14.1 [11]	15	26.7 [4]	0.809	83	25.3 [21]	28	35.7 [10]	0.508
Family history of premature cardiovascular disease, % [n]	76	26.3 [20]	13	23.1 [3]	0.949	74	45.9 [34]	25	44.0 [11]	0.760
Statin treatment, % [n]	78	55.1 [43]	15	33.3 [5]	0.028	83	100 [83]	28	100 [28]	-
Sex, women % [n]	78	56.4 [44]	15	26.7 [4]	0.056	83	67.5 [56]	28	60.7 [17]	0.360
Tendon xanthomas, % [n]	78	0.0 [0]	15	0.0 [0]	1.000	78	39.7 [31]	24	37.5 [9]	0.870

Continuous data expressed as mean (SD); categorical data are expressed as percentages [count]. LDLc: low-density lipoprotein cholesterol. P- values from linear and logistic regressions based on generalized linear models (GLM), adjusted for sex and age
